# Modulation of Macrophage Polarization and HMGB1-TLR2/TLR4 Cascade Plays a Crucial Role for Cardiac Remodeling in Senescence-Accelerated Prone Mice

**DOI:** 10.1371/journal.pone.0152922

**Published:** 2016-04-12

**Authors:** Vengadeshprabhu Karuppagounder, Vijayasree V. Giridharan, Somasundaram Arumugam, Remya Sreedhar, Suresh S. Palaniyandi, Prasanna Krishnamurthy, Joao Quevedo, Kenichi Watanabe, Tetsuya Konishi, Rajarajan A. Thandavarayan

**Affiliations:** 1 Department of Clinical Pharmacology, Faculty of Pharmaceutical Sciences, Niigata University of Pharmacy and Applied Life Sciences, Niigata 956–8603, Japan; 2 Department of Psychiatry and Behavioral Sciences, The University of Texas Health Science Center at Houston, TX 77054, United States of America; 3 Division of Hypertension and Vascular Research, Henry Ford Health System, Detroit, MI 48202, United States of America; 4 Department of Cardiovascular Sciences, Houston Methodist Research Institute, Houston, TX 77030, United States of America; 5 Basic studies on second generation functional foods, NUPALS Liaison R/D promotion division, Higashijima 265–1, Akiha-ku, Niigata, Japan; 6 Changchun University of Chinese Medicine, Bosuo Road #1035 Jingyue Economic Development District, Changchun, RP China; Georgia Regents University, UNITED STATES

## Abstract

The aim of this study was to investigate the role of macrophage polarization in aging heart. Macrophage differentiation is pathogenically linked to many inflammatory and immune disorders. It is often preceded by myocardial inflammation, which is characterized by increased cardiac damage and pro-inflammatory cytokine levels. Therefore, we investigated the hypothesis that senescence accelerated-prone (SAMP8) mice cardiac tissue would develop macrophage polarization compared with senescence-resistant control (SAMR1) mice. Both SAMP8 and SAMR1 mice were sacrificed when they became six month old. We evaluated, histo-pathological changes and modifications in protein expression by Western blotting and immuno-histochemical staining for M1 and M2 macrophage markers, high mobility group protein (HMG)B1 and its cascade proteins, pro-inflammatory factors and inflammatory cytokines in cardiac tissue. We observed significant upregulation of HMGB1, toll-like receptor (TLR)2, TLR4, nuclear factor (NF)κB p65, tumor necrosis factor (TNF)α, cyclooxygenase (COX)2, interferon (IFN)γ, interleukin (IL)-1β, IL-6 and M1 like macrophage specific marker cluster of differentiation (CD)68 expressions in SAMP8 heart. In contrast, M2 macrophage specific marker CD36, and IL-10 expressions were down-regulated in SAMP8 mice. The results from the study demonstrated that, HMGB1-TLR2/TLR4 signaling cascade and induction of phenotypic switching to M1 macrophage polarization in SAMP8 mice heart would be one of the possible reasons behind the cardiac dysfunction and thus it could become an important therapeutic target to improve the age related cardiac dysfunction.

## Introduction

Aging is characterized by increase in the anticipation of death overtime associated with unique changes in phenotype [1]. By 2030, approximately 20% of the population will be older and incident coronary heart disease is projected to increase by approximately 26% in 2040 [2]. Rising of aging population is accompanied by a sharp increase in the prevalence of age-associated chronic diseases ranging from cardiovascular diseases (CVD), Alzheimer’s disease and cancer to metabolic syndrome [3]. CVD includes coronary artery disease, hypertension, and chronic heart failure and they are the leading cause of death worldwide. Reports stated that approximately 85% of CVD patients die at 65 years or older. Similarly, aging is associated with a dramatic growth in the prevalence of high blood pressure and chronic heart failure [4]. Since advancing age is such a crucial risk factor for the progress of this pathophysiological conditions, we used the senescence-accelerated prone mice (SAMP8), a murine model of spontaneous senescence that mimics many common geriatric disorders in the human population [5].

Macrophages are hallmarked by phenotypic heterogeneity and are widely distributed in different tissues and potent immune regulators [6]. The macrophage differentiations are divided into two phenotypes: classical M1 and alternative M2. Classically activated M1 macrophages have long been recognized to be induced by lipopolysaccharide (LPS) or interferon (IFN)γ or cytokines. Activated M1 macrophages secrete large amounts of pro-inflammatory mediators such as high mobility group protein (HMG)B1, which is believed to contribute to the inflammation. HMGB1 has been reported to transduce its signals by interacting with three important receptors such as receptor for advanced glycation end products (RAGE) and toll like receptor (TLR)2/TLR4. Activated TLR2/TLR4 and RAGE signaling induces nuclear factor kappa (NFκ)B and extracellular signal-regulated kinases (ERK)1/2 signaling, which triggers cytokine production [7]. On the far side, alternatively activated M2 macrophages are stimulated by interleukin (IL)-4 and IL-13, which act to restrict these inflammatory responses through IL-10 secretion and mediate tissue repair [8]. On the basis of the written reports, we hypothesized and attempted to demonstrate the modulation of M1 macrophage polarization and HMGB1-TLR2/TLR4 cascade signaling plays a significant part in the pathogenesis of cardiac dysfunction with aging, in SAMP8 mouse model.

## Methods and Methods

### Materials

All the reagents and chemicals were of analytical grade and purchased from Sigma or Wako, Tokyo, Japan, until mentioned otherwise.

### Experimental design

SAMP8 and senescence-resistant control (SAMR1) male mice were provided by Japan SLC Inc., and were maintained individually (because of their aggressive behavior) under standard conditions (temperature 23 ± 1°C, humidity 50–60%, 12:12-h light-dark cycle, lights on at 7:00 a.m.), with food in the form of dry pellets and tap water available ad libitum throughout the study. Both SAMP8 and SAMR1 mice (n = 8 each) were sacrificed when they became 24 weeks old and their heart tissues were harvested for semi-quantitative immuno-blotting and immuno-histochemical studies. The half of the ventricle was immediately frozen in liquid nitrogen for subsequent protein extraction assays. The remaining excised heart was cut into about 2 mm thick transverse slices and fixed in 10% formalin. All animal protocols used in this study were approved by the Institutional Review Board at Niigata University of Pharmacy and Applied Life Sciences.

### Protein analysis by Western blotting

Protein samples were subjected to sodium dodecyl sulfate (SDS) polyacrylamide gel electrophoresis and then transferred to nitrocellulose membranes [9]. Membranes were blocked with 5% bovine serum albumin (BSA) in Tris-buffered saline (TBS) with 0.1% tween (TBST) and incubated individually with the following antibodies: Antibodies against HMGB1, RAGE, phospho (p)-NFκB p65, NFκB p65, cyclooxygenase (COX)2, tumor necrosis factor (TNF)α, TNF receptor (TNFR)1, IL-1β, IL-6, TLR4, TLR2, p- (ERK)1/2, ERK1/2, IFNγ, and cluster of differentiation (CD)68. All the antibodies were purchased from Santa Cruz Biotechnology, Inc. (Santa Cruz, CA, USA) or Cell Signaling Technology, Inc. (Danvers, MA, USA) and used at a dilution of 1:1000. After washing for three times with TBST, the membranes were incubated with appropriate horseradish-peroxidase (HRP) conjugated secondary antibodies for 1 h at room temperature. Further, the membranes were washed three times with TBST and then developed using a chemiluminescence detection system (Amersham Biosciences, Buckinghamshire, UK). The blots were scanned and the signals were quantified by densitometric analysis using Image Studio Digits ver. 4 (Superior Street, Lincoln, Nebraska, USA). Glyceraldehyde-3-phosphate dehydrogenase (GAPDH) served as control protein.

### Immunohistochemical analysis

Formalin-fixed, paraffin-embedded heart tissue sections were used for immunohistochemical staining of CD36 or IL-10. After deparaffinization and hydration, the slides were washed in TBS (10 mM/1 Tris HCl, 0.85% NaCl, pH 7.2). Endogenous peroxidase activity was quenched by incubating the slides in 0.3% H_2_O_2_ in methanol. The slides were blocked with either 10% goat serum or rabbit serum for 1 h at room temperature and washed three times with TBS for 5 min each. After overnight incubation with the primary antibody, either rabbit polyclonal anti-CD36 or goat polyclonal anti-IL-10 (diluted 1:100) (Santa CruZ Biotechnology, Inc., CA, USA), at 4°C, the slides were washed in TBS and then HRP conjugated secondary antibody was added and further incubated at room temperature for 45 min. The slides were rinsed in TBS and incubated with diaminobenzidine tetra hydrochloride as the substrate and counterstained with hematoxylin. Brown colored CD36 and IL-10 immunopositive cells were counted using a 40X objective [10].

### Statistical analysis

Data are presented as mean±standard error of mean (SEM) and were analyzed using two-tailed t-test. A value of p<0.05 was considered statistically significant. For statistical analysis, GraphPad Prism 5 software (GraphPad Software. Inc., San Diego, CA, USA) was used.

## Results

### Cardiac expression of M2-like macrophage phenotype in SAMP8 mice

**Fig 1:**
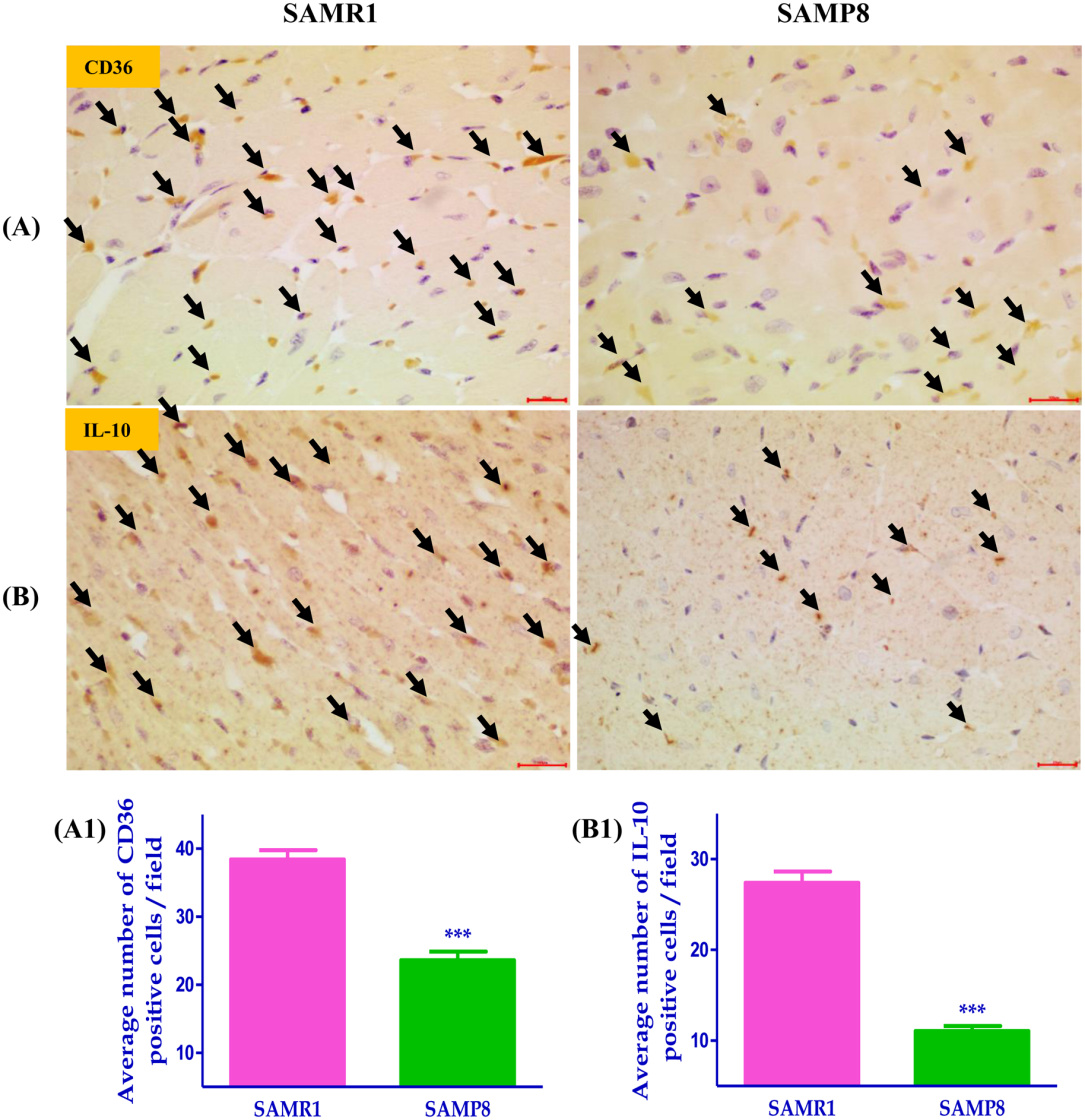
Immunohistochemical staining showing (A-A1) CD36 positive cells and its quantification data, (B-B1) IL-10 positive cells and its quantification data (scale bar = 20μm). Each bar represents mean±SEM. SAMR1, age-matched normal senescence accelerated resistant controls mice; SAMP8, senescence accelerated prone mice; ***p<0.001 vs SAMR1 (Two-tailed t-test).

We examined whether aging would decrease M2 phenotype in SAMP8 mice, as aging impairs macrophage polarization into M1 and M2 subtypes, and a decrease in cardiac M2 macrophages [11]. As shown in Fig 1A-A1 and 1B-B1, immunohistochemical staining showed that the M2 phenotype such as CD36, and IL-10 positive cells were significantly down-regulated in the heart section of SAMP8 mice, when compared with SAMR1 mice.

### Cardiac expression of M1-like macrophage phenotype in SAMP8 mice

**Fig 2:**
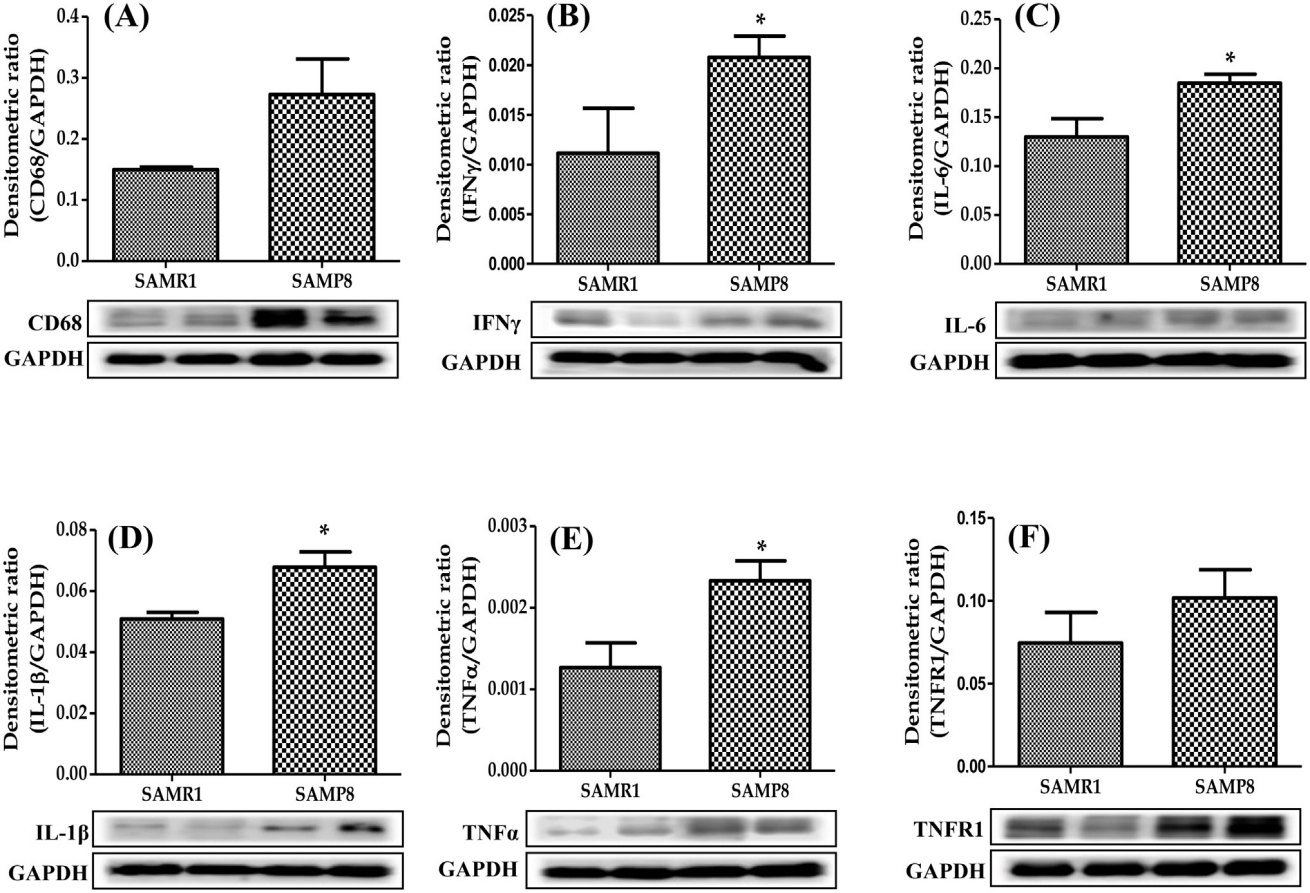
Western blots show specific bands for the expression of (A-F) CD68, IFNγ, IL-6, IL-1β, TNFα, and TNFR1 (ratio relative to that of GAPDH). Each bar represents mean±SEM. SAMR1, age-matched normal senescence accelerated resistant controls mice; SAMP8, senescence accelerated prone mice. *p<0.05 vs SAMR1 (Two-tailed t-test).

**Fig 3:**
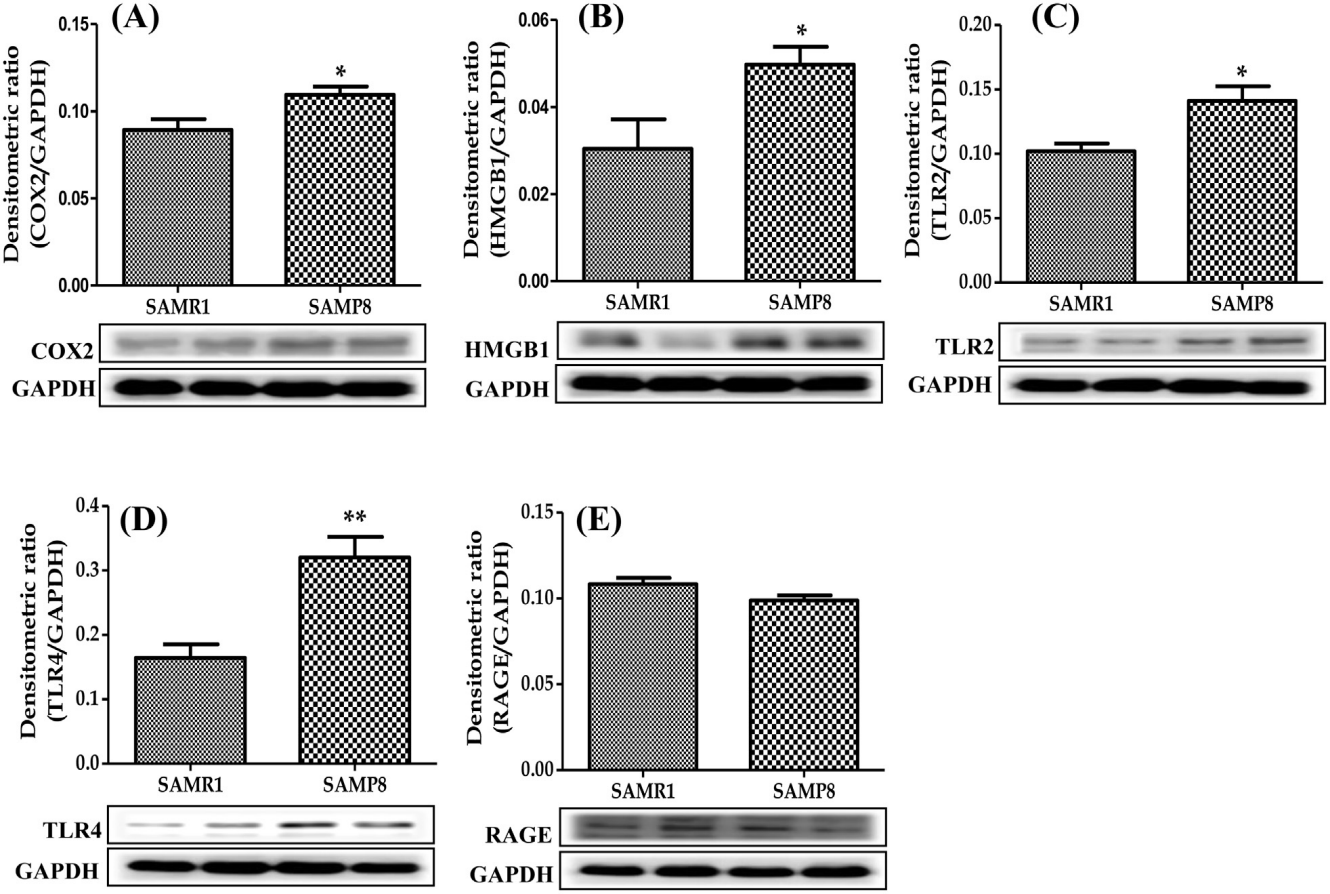
Western blots show specific bands for the expression of (A-E) COX2, HMGB1, TLR2, TLR4 and RAGE (ratio relative that of GAPDH). Each bar represents mean±SEM. SAMR1, age matched normal senescence accelerated resistant controls mice; SAMP8, senescence accelerated prone mice. *p<0.05 and **p<0.01 vs SAMR1 (Two-tailed t-test).

Cytokines and pro-inflammatory factors can stimulate macrophage polarization to M1 phenotype. Thus, we performed Western blot analysis to evaluate the specific M1 macrophage marker CD68 and other inflammatory and pro-inflammatory factors such as IFNγ, IL-6, IL-1β, TNFα, TNFR1 and COX2 in the heart homogenates. The protein expression of all these factors were dramatically increased in SAMP8 mice compared with those in control SAMR1 mice. (Figs 2A–2F and 3A).

### Cardiac expression of HMGB1, TLR2, TLR4 and RAGE in SAMP8 mice

M1 type macrophages secrete HMGB1 or its activate release into extracellular space and the released HMGB1 binds to RAGE, TLR2 and TLR4, leading to the activation of cell migration dependent signaling [12, 13]. In the present study, Western blot analysis data showed that the protein expression of HMGB1, TLR2 and TLR4 were significantly increased in SAMP8 mice compared with those in SAMR1 mice. However, there was no significant difference in RAGE expression was observed between SAMP8 and SAMR1 group (Fig 3B and 3E).

### Cardiac expression of p-ERK1/2 and p-NFκB p65 in SAMP8 mice

**Fig 4:**
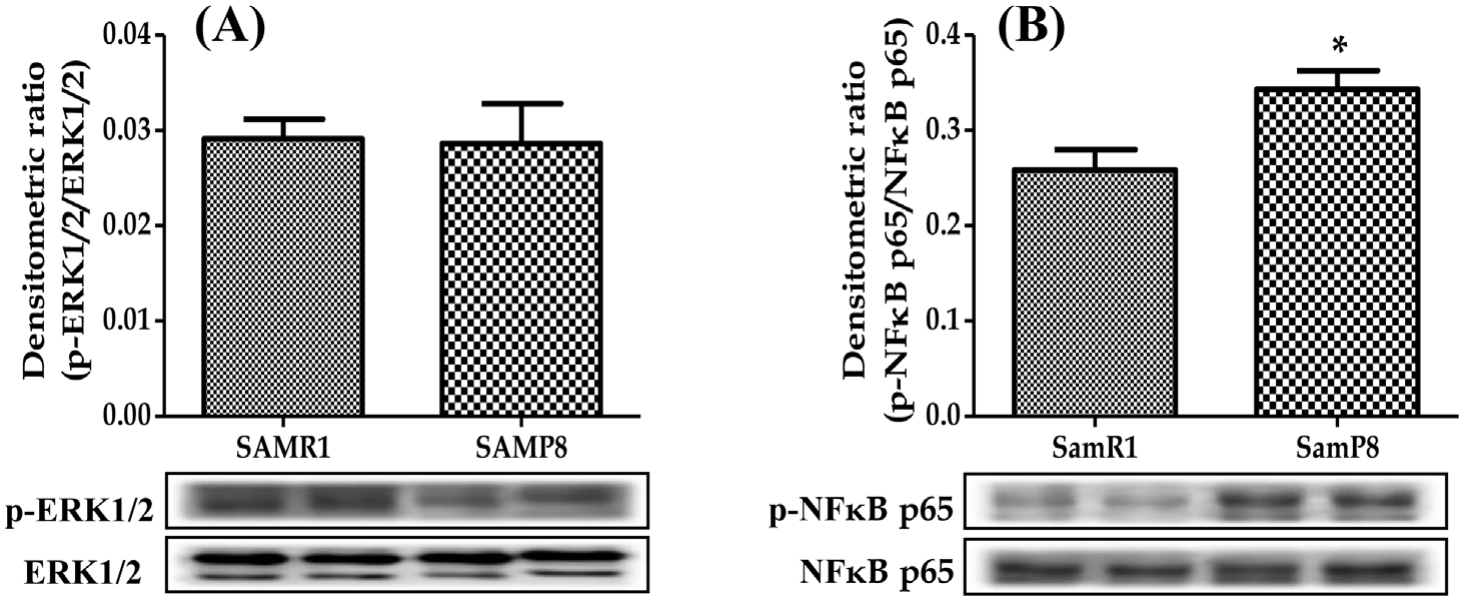
Western blot shows specific bands for the expression of (A) p-ERK1/2 (ratio relative to that of ERK1/2), (B) p-NFκB p65 (ratio relative to that of NFκB p65). Each bar represents mean±SEM. SAMR1, age-matched normal senescence accelerated resistant controls mice; SAMP8, senescence accelerated prone mice. *p<0.05 vs SAMR1 (Two-tailed t-test).

Binding of HMGB1 to TLR2 and TLR4 initiates NFκB p65 and ERK1/2 signaling pathway. Thus, we performed Western blot analysis to evaluate the protein expressions of ERK1/2 and NFκB p65 in the heart homogenates. The protein expression of p-NFκB was significantly increased in SAMP8 mice compared with those in SAMR1 mice. In contrast, p-ERK1/2 expression was unchanged between SAMP8 and SAMR1 group (Fig 4A and 4B).

## Discussion

In our study, the most important findings were as follows (a) aging heart enhances M1-like macrophage phenotype, HMGB1 cascade and inflammatory cytokine levels in SAMP8 mice. (b) Aging suppresses anti-inflammatory gene expression by switching M1 phenotype, leading to decreased CD36 and IL-10 protein levels in SAMP8 mice heart.

The SAMP8 mouse was one of the accelerated senescence strains that spontaneously developed from AKR/J series of breeding pairs [14]. Advanced age increases the chance of exposure to cardiovascular risk factors [15]. The SAMP8 mice showed enhanced diastolic dysfunction similar to those seen in humans with aging [16]. In addition, age-dependent accumulations of RNA and DNA damages were observed in SAMP8 mice heart [17]. It has likewise been indicated that SAMP8 mice develop myocardial damage and induce pro-inflammatory and inflammatory cytokines [18]. Although several studies strongly indicate that the pro-inflammatory factors and inflammatory cytokines are known to involve in the pathogenesis of cardiovascular diseases in aging, there were no reports about the potential mechanism of M1 and M2 macrophage polarization in aging heart. In the present study, we show for the first time that the macrophage polarization and cardiac inflammation in SAMP8 mice through activation of the M1 macrophage polarization and HMGB1-TLR2/TLR4 signaling pathways.

Macrophages show significant heterogeneity in function, as local environmental factors shape their properties and activation state. Broadly, macrophages have been characterized as two distinct polarization states, “classical” or M1 phenotype and M2 phenotype, mirroring the T helper cell (Th)1-Th2 polarization represent two extremes of an effective changing state of macrophage activation [19]. The M1 phenotype activation is closely linked with tissue destruction and inflammation [20]. However, it is well proved and was previously described that the M1 marker CD68 positive macrophages were accumulated in human ischemic heart and idiopathic dilated cardiomyopathy and caused the release of M1 macrophage associated pro-inflammatory factors [21] and cytokines such as IFNγ, IL-6, IL-1β, and TNFα [22]. Activated pro-inflammatory factors and cytokines play important roles in cardiac inflammation [22]. Moreover, pro-inflammatory cytokines such as IL-1β and IL-6 levels were highly augmented in the heart of aging mice [23]. In the present study, many CD68 positive cells were accumulated in the heart with upregulation of pro-inflammatory factors and cytokines such as IFNγ, IL-6, IL-1β, TNFα, TNFR1 and COX2 expression in aging SAMP8 mice. These data clearly suggest that the aging modulates the macrophages into a state with increased capacity to enhance pro-inflammatory factors and cytokines associated with the M1 like phenotype in the heart.

HMGB1, a chromosomal protein with high electrophoretic mobility, has distinctive roles depends upon the cell location: in the nucleus, it promotes nuclear transcription, replication, recombination and DNR repair [24]. On other side, translocation of HMGB1 from nucleus to cytoplasm or extracellular phase makes it as a pro-inflammatory cytokine [25]. Importantly, recent studies showed that, LPS induced cardio-myocytes produce and secrete HMGB1, which mediates myocardial dysfunction [26]. In addition, pressure overload encourages the HMGB1 levels and mediate inflammation in ischemic-reperfused heart. A similar scenario has been described in the diabetic heart, the activated HMGB1 promote myocardial fibrosis and heart dysfunction [27]. In our study, we also found that HMGB1 expression was upregulated in SAMP8 aging mice heart. Hence, it is possible to suggest that in aging, M1-like macrophage might activate HMGB1 and it can act as an inflammatory cytokine leading to heart inflammation.

Various lines of evidence confirm that the HMGB1 mediated inflammatory reaction could be mediated through TLR2, TLR4 and RAGE. HMGB1 binding to TLR2, TLR4 and RAGE results in downstream danger signaling activation [28]. TLRs are regulated by pattern recognition receptors and prominently expressed in macrophages. TLR2 and TLR4 are the most broadly studied receptors in cardiovascular injury [29]. Consistently, TLR2 and TLR4 knockout mice have a reduced infarct size, improved cardiac function and myocardial inflammation [30, 31]. Other studies showed that RAGE in the primary binding receptor for HMGB1 and it mediates cytokine activity; leading to promote myocardial injury in ischemic reperfusion model [32]. Impressively, in our study the TLR2 and TLR4 expressions were significantly upregulated in SAMP8 mice heart. In contrast, RAGE protein expression was not changed in SAM8 mice when compared with SAMR1 mice. It is possible to hypothesize that HMGB1 might interact with TLR2 and TLR4 to produce inflammation in aging heart.

The interaction of HMGB1-TLR2/TLR4 leads to trigger ERK1/2 and NFκB signaling pathway [33]. Increasing evidence suggests that activation of ERK seems a causal factor for cardiac inflammation by HMGB1 [34], and phosphorylated ERK has been associated with macrophage polarization with aortic aneurysm and inflammatory process [35]. In the present study, no change in the ERK expression was observed between SAMP8 and SAMR1 mice. This observation brings up a number of inquiries about the role of ERK in the aging heart, and further studies are required to elucidate the precise mechanism of ERK1/2 protein during aging heart. HMGB1-TLRs interaction can activate the NFκB signaling pathway, and activated NFκB plays a significant part in inflammatory phenotypic changes in endothelial dysfunction and various heart diseases [36]. Recent experimental data suggests that the NFκB signaling seems to be involved in the entropic aging process and is likely responsible for the increased production of pro-inflammatory factors and cytokines with aging [37]. In this study, we found that NFκB expression was upregulated in SAMP8 mice heart. This finding may be explained by HMGB1-TLR2/TLR4 binding induced NFκB signaling pathway leading to the inflammatory response in aging heart.

On the other side, the anti-inflammatory (M2 phenotype) or alternatively activated macrophages are stimulated by IL-4, IL-10, immune complexes, and glucocorticoids [20]. Activated M2 macrophage polarization suppresses M1 macrophage polarization, pro-inflammatory cytokines and promotes tissue repair. The anti-inflammatory proteins such as IL-10 and CD36 attenuate the expression of pro-inflammatory molecules in macrophages [38]. Furthermore, IL-10 cytokine alleviates inflammation in atherosclerosis model [39]. In chronic conditions, the functions of M2 phenotype may differ in aging mice. Nevertheless, aging has been linked with enhanced M1 macrophage polarization and suppressed M2 phenotype [40]. Interestingly, in our study, the M2-like macrophage marker CD36 and anti-inflammatory marker IL-10 levels were significantly downregulated in SAMP8 aging mice heart. These findings demonstrated that the suppression of myocardial M2 phenotype due to the aging mediated switching to M1 macrophage polarization and increased pro-inflammatory factors and cytokine levels, which leads to cardiac dysfunction and inflammation. However, mechanism behind the how switching of M2 to M1 phenotype in the heart during aging is unclear.

In conclusion, considering all these findings, it is suggested that the aging induced M1 phenotype might have differential consequences on HMGB1 expression and its cascade signaling, such as stimulation of TLR2, TLR4, p-NFκB 65, pro-inflammatory factors and cytokines in addition to decreased M2 phenotype such as CD36, and IL-10 expressions in aging heart. Modulation of M1 macrophage polarization and the HMGB1-TLR2/TLR4 cascade will eventually lead to the decrease M2 phenotype as well as myocardial dysfunction during aging. From these results we can suggest that the drugs which modulate the M1 macrophage polarization and HMGB1-TLR2/TLR4 cascade could become a potential therapy for the amelioration of aging related cardiac inflammation.
